# Oropouche Virus (OROV) in Pregnancy: An Emerging Cause of Placental and Fetal Infection Associated with Stillbirth and Microcephaly following Vertical Transmission

**DOI:** 10.3390/v16091435

**Published:** 2024-09-09

**Authors:** David A. Schwartz, Pradip Dashraath, David Baud

**Affiliations:** 1 Perinatal Pathology Consulting, 490 Dogwood Valley Drive, Atlanta, GA 30342, USA; 2 Division of Maternal-Fetal Medicine, Department of Obstetrics and Gynecology, Yong Loo Lin School of Medicine, National University of Singapore, 1E Kent Ridge Road, Singapore 119228, Singapore; pradip_dashraath@nuhs.edu.sg; 3 Materno-Fetal & Obstetrics Research Unit, Department Woman-Mother-Child, Lausanne University Hospital, Centre Hospitalier Universitaire Vaudois, 1011 Lausanne, Switzerland; david.baud@chuv.ch

**Keywords:** Oropouche virus, OROV, epidemic, arbovirus, vertical transmission, Bunyavirus, congenital infection, microcephaly, stillbirth, pregnancy, placenta, imported infection, miscarriage, fetal demise, Brazil

## Abstract

Oropouche virus (OROV) is an emerging arbovirus endemic in Latin America and the Caribbean that causes Oropouche fever, a febrile illness that clinically resembles some other arboviral infections. It is currently spreading through Brazil and surrounding countries, where, from 1 January to 1 August 2024, more than 8000 cases have been identified in Bolivia, Brazil, Columbia, and Peru and for the first time in Cuba. Travelers with Oropouche fever have been identified in the United States and Europe. A significant occurrence during this epidemic has been the report of pregnant women infected with OROV who have had miscarriages and stillborn fetuses with placental, umbilical blood and fetal somatic organ samples that were RT-PCR positive for OROV and negative for other arboviruses. In addition, there have been four cases of newborn infants having microcephaly, in which the cerebrospinal fluid tested positive for IgM antibodies to OROV and negative for other arboviruses. This communication examines the biology, epidemiology, and clinical features of OROV, summarizes the 2023–2024 Oropouche virus epidemic, and describes the reported cases of vertical transmission and congenital infection, fetal death, and microcephaly in pregnant women with Oropouche fever, addresses experimental animal infections and potential placental pathology findings of OROV, and reviews other bunyavirus agents that can cause vertical transmission. Recommendations are made for pregnant women travelling to the regions affected by the epidemic.

## 1. Introduction

During the past decade, it has been dramatically reinforced that infectious diseases must no longer remain confined to their endemic areas. The global spread of mpox (monkeypox) Clade IIb beginning in 2022 from Africa, the COVID-19 pandemic that began in late 2019 in China, and the Zika virus epidemic starting in Brazil in 2015 are all examples that ultimately affected the world population. The vulnerability of pregnant women and the fetus to emerging and re-emerging viruses, including mpox, SARS-CoV-2, Zika, and Ebola viral infections [1,2,3,4,5,6], has been a significant public health challenge during these epidemics. Mpox (monkeypox) is especially of medical concern due to its recent global spread, its ability to infect the fetus via transplacental transmission, and the recent reports of a 50 percent fetal death rate among infected mothers during the current outbreak in the Democratic Republic of the Congo [7,8]. A common feature of all of these outbreaks was the frequency with which they infected pregnant women, causing transplacental fetal infection, disability, stillbirth, and perinatal death. This communication describes a little-known tropical arboviral infection termed Oropouche fever that has reached epidemic proportions in Latin America since 2023, becoming the newest emerging viral disease responsible for congenital vertical transmission and fetal death. 

## 2. Oropouche Virus (OROV)

The most recent global infectious disease outbreak of concern is the result of the Oropouche virus (OROV), a previously neglected tropical zoonosis [9,10]. OROV is an emerging arthropod-borne Orthobunyavirus circulating in Central America, South America, and the Caribbean. It is most frequently encountered in Brazil, where it is the second most common arthropod-borne viral infection after dengue virus [11]. The virus was initially described in 1955 in the blood of a symptomatic forest worker in Trinidad and Tobago, acquiring its name from the village of Vega de Oropouche, where it was first found [12]. OROV belongs to the family Peribunyaviridae and contains a three-part single-stranded negative-sense RNA genome consisting of three segments: the small (S) segment encoding for nucleotides (nucleocapsid protein N and the nonstructural (NS) protein NSs); the medium (M) segment encoding glycoproteins (Gn, Gc, and NSm protein); and the large (L) segment encoding for L protein and an RNA-dependent RNA polymerase [11]. OROV has four major genotypes (I to IV), which are endemic in varying but partially overlapping regions of Latin America. Because the genome of bunyaviruses such as OROV is segmented, reassortment may occur between its many members. This inherent capability for gene rearrangement is an important feature that creates genetic diversity and accounts for the recombinant viruses within the species, such as the Madre de Dios virus in northeast Venezuela and southeast Peru, Perdões virus in southeast Brazil, and the Iquitos virus in northeast Peru. There is preliminary evidence that the current 2022–2024 OROV epidemic that began in the Brazilian Amazon basin may result from a novel viral lineage reassortment that emerged in the Central region of Amazonas State [13,14].

## 3. Epidemiology

The Oropouche virus has been known to cause human infections in some jungle and forested regions of South America for several decades. Still, scant attention has been paid to it; thus, little is known about the disease. OROV naturally occurs in two cycles: a sylvatic cycle that includes specific vertebrate hosts as a natural reservoir (pale-throated sloths (*Bradypus tridactylus*), non-human primates, marsupials, and birds) and mosquitoes and an urban cycle that consists of humans as amplifying hosts and arthropod vectors including biting midges (*Culicoides paraensis*) and mosquitoes (*Aedes serratus* and *Culex quinquefasciatus*). There is currently no evidence that OROV is transmissible person to person except for vertical transmission during pregnancy, and it remains unknown if OROV can be transmitted by body fluids [15,16].

Since the initial isolation of the virus, more than 30 human outbreaks have been reported in Bolivia, Brazil, Colombia, Ecuador, French Guiana, Panama, and Peru, with at least half a million persons infected [17]. In Brazil, OROV is one of the most common viruses to infect humans. However, the number of affected persons can only be estimated because of multiple factors, including lack of surveillance and reporting, prevalence in remote locations such as the Amazon region making case identification difficult, poor availability of diagnostic reagents, and the similarity of Oropouche fever symptoms with other circulating arboviruses. 

Beginning in late 2023, OROV infections have rapidly increased in South America. The most significant number occurred in Brazil, where there were 7044 confirmed cases of Oropouche fever in 2024, greater than 8 times the number that occurred the previous year [18]. Between 1 January and 1 August 2024, a total of 8078 new cases of OROV infection were reported in Brazil, Bolivia, Peru, Colombia, and Cuba [14,19]. In Latin America, OROV is occurring in regions where it had not been endemic, including the first descriptions of the infection in Cuba [20]. OROV disease is also spreading through international travel outside of Latin America and the Caribbean. As of 27 August 2024, OROV infection has been confirmed in 21 U.S. travelers who acquired the disease while in Cuba [21]. OROV cases have also been imported from Latin America into Europe [22] as of July 2024, including 12 cases in Spain, 5 in Italy, and 2 in Germany; 18 cases originated in Cuba and 1 in Brazil. 

The reasons for the sudden emergence of Oropouche fever in Brazil and surrounding countries are both complex and unclear. In addition to the evidence for genetic reassortment causing a novel strain of OROV [13,14], there are also factors related to the environment and natural history of the infection. Similar to other vector-borne diseases dissemination is controlled by the changing interplay of climate, land use, deforestation, movements and migrations of humans and animals, ecology of the vertebrate hosts and insect vectors, and human behavior patterns [14].

## 4. Clinical Features of Oropouche Fever

OROV causes Oropouche fever, sometimes called sloth fever, which is symptomatic in approximately 60% of infected persons and presents with abrupt onset of a febrile illness, headache, arthralgia, myalgia, nausea, and vomiting. Other symptoms include diarrhea, abdominal pain, retro-orbital pain, photophobia, dizziness, conjunctival injection, and a maculopapular rash that starts on the trunk and spreads to the limbs [15]. Hemorrhagic symptoms occur less frequently and include cutaneous petechiae, melena, menorrhagia, gingival bleeding, and epistaxis [23]. Clinical illness from Oropouche fever can be confused with other endemic arboviral infections in Latin America, including dengue, chikungunya, Zika, and Mayaro. The disease is typically mild and symptoms generally resolve in less than one week, but recurrences can occur in 60 to 70% of cases. One of the most severe manifestations of Oropouche fever is central nervous system infection occurring in approximately 4% of cases, including aseptic meningitis and meningoencephalitis [24,25,26]. The risk factors for developing central nervous system infection with OROV include immunocompromised adults and children and conditions resulting in disruption of the blood–brain barrier [27]. The exact mechanism for central nervous system infection is unknown. Because OROV can be detected in the bloodstream during the early phases of infection, it most likely reaches brain tissue as a result of viremia [25]. The incubation period of OROV is variable between 3 and 10 days after being bitten by an infected arthropod [15]. Following this, the infected individuals have high levels of OROV viremia, which declines to levels of 44% of maximum on day 4 and 23% by day 5 of illness, which is an important factor in the development of placental infection in pregnant patients [28].

Despite the large numbers of prior infections and descriptions of central nervous system infection, there had been no fatalities reported to occur from Oropouche fever until the 2023–2024 epidemic, when fatalities were reported from the State of Bahia in July 2024 [29]. Two nonpregnant women less than 30 years of age having no comorbidities developed acute Oropouche fever symptoms that included fever, myalgia, retro-orbital pain, and headache. Their illness progressed rapidly to respiratory failure, hypotension, bleeding, abdominal pain, and, eventually, death [14,29].

## 5. OROV in Pregnancy, the Placenta, and Fetus

Recent reports from the ongoing 2023–2024 Oropouche fever epidemic indicate that this is the newest arbovirus to affect pregnancy outcomes adversely. However, in retrospect, there had been previous evidence suggesting that OROV was causing pregnancy losses during an outbreak that occurred in Manaus, Brazil, between 1980 and 1981 [30,31]. During this outbreak, Oropouche fever was identified in nine pregnant women, among whom two had first-trimester miscarriages. However, the association between OROV and fetal loss was not further investigated until the present epidemic. Beginning in July 2024, there has been accumulating evidence obtained from multiple reports of pregnant women with Oropouche fever in Brazil having miscarriages, stillbirths, and infants with neonatal microcephaly to support the conclusion that this bunyavirus has become the most recent emerging virus to cause maternal–fetal transplacental infection and congenital infection. On 12 July 2024, the Brazilian authorities reported to WHO/PAHO the occurrence of a presumptive case of maternofetal (vertical) transmission of OROV [32] in Pernambuco State. The mother developed Oropouche fever symptoms, including headache, epigastric pain, and fever, on 24 May 2034. She had been in close contact with a person with Oropouche fever in the territory. Samples collected from the woman on 3 June had a reactive response for dengue and chikungunya viruses by ELISA-IgM testing (ELISA-IgM). At 30 weeks gestation, no fetal movement was noted, and an intrauterine fetal demise was confirmed. Following delivery and performance of a fetal autopsy, molecular analysis performed at the Evandro Chagas Institute confirmed OROV infection with positive RT-PCR testing of the umbilical cord and placenta, as well as multiple fetal somatic organs, including the brain, kidneys, spleen, lungs, liver, and heart. Fetal tissue specimens were negative for molecular detection of other arboviruses, including Zika, dengue, Mayaro, and chikungunya. These findings are characteristic of a transplacental maternal–fetal viral infection, similar to the patterns of intrauterine transplacental transmission in such recent emerging viruses causing stillbirth such as mpox (monkeypox virus) infection in DR Congo [8,33,34] and SARS-CoV-2 [35,36,37]. A second fetal demise suspected to be due to Oropouche fever occurred in a 33-year-old woman who had clinical features of OROV infection during the first trimester, developed uterine hemorrhage, and had a miscarriage on 27 June 2024, at 8 weeks gestation. Maternal serum collected on 12 June was positive for OROV by PCR, reactive for dengue using IgM ELISA, and negative for other viral pathogens [18]. At least three other fetal deaths associated with maternal OROV infection are under investigation, and more detailed analyses are ongoing [14]. On 17 July, PAHO issued an Epidemiological Alert regarding the association of OROV with vertical transmission and congenital fetal anomalies [32].

The Brazilian Ministry of Health reported evidence of an association between Oropouche fever and congenital microcephaly. Analysis of cerebrospinal fluid (CSF) and serum samples collected for arbovirus investigation identified four neonates having microcephaly; IgM antibodies against OROV were identified from serum in infants at day of life (DOL) 1 and 27 and, in CSF, in two newborns at DOL 1 and DOL 27. The serum samples from all four infants tested negative for West Nile, Zika, dengue, chikungunya, and dengue viruses [14,38]. Direct pathology evidence for the association of OROV with vertical transmission was identified following an autopsy of a liveborn infant with microcephaly and other congenital malformations who had been delivered to a mother with skin rash and fever during the second month of pregnancy and who tested positive for OROV during the postpartum period [39]. Fetal ultrasonography at 33 weeks’ gestation was abnormal, and MRI confirmed microcephaly, severe ventriculomegaly, thinning of the brain parenchyma, agenesis of the corpus callosum, and oligohydramnios. The infant had IgM antibodies against OROV after delivery at 36 weeks’ gestation. Following death at DOL 47, OROV was identified by molecular testing in cerebrospinal and pleural fluids and in fetal organs, including kidneys, lungs, and brain [39,40]. These findings are all consistent with maternal OROV viremia resulting in transplacental infection, termed hematogenous transmission.

It should be no surprise that placental and fetal somatic organ infections have occurred following maternal infection during the OROV outbreak. Multiple bunyaviruses have a tropism for fetal and placental tissues, causing abortions, fetal losses, and multiple congenital deformities in pregnant livestock. Intrauterine infections from related orthobunyavirus members of the Simbu serogroup can cause transplacental infection in cattle and other ruminants, including such severe fetal anomalies as arthrogryposis, cerebellar hypoplasia, and hydranencephaly, accompanied by microscopic neuropathology, including spongy degeneration and nonsuppurative encephalomyelitis [38,41]. The Batai virus (BATV) is another example of a bunyavirus that causes premature births, abortions, and congenital defects in livestock [42]. Cache Valley virus (CVV) is an orthobunyavirus that causes spontaneous abortions, fetal malformations, infertility, and congenital abnormalities in sheep and goats; it has been reported to infect humans [43,44]. In particular, OROV appears to have a specific neurotropism [45,46], consistent with the recent reports of microcephaly in human fetuses. Experimental infection of nonpregnant golden hamsters with OROV induces viremia, hepatitis and meningoencephalitis, microglial activation, and viral antigen in neurons and hepatocytes using immunohistochemistry [45]. Suckling mice infected with OROV develop central nervous system infections in which neurons are the major target cells of viral infection and in which viral replication occurs, as well as astrocyte activation, gliosis in the brain and spinal cord, and apoptotic neurons [46]. 

The reports of adverse pregnancy outcomes that have recently been associated with OROV infection in Brazil, including miscarriage, stillbirth, and congenital anomalies, have some overlapping features of the placental and fetal effects of such recent emerging viruses as Zika virus, SARS-CoV-2, MPXV, and Ebola virus (Table 1). In examining this table, the perinatal outcomes data available thus far show that OROV has features most similar to ZIKV, another arbovirus, despite being classified in a different phylum. 

## 6. Placental Pathology

An essential component of additional research into the effects of OROV on pregnancy and fetal development should include pathological examination of placental and fetal tissues and the use of nucleic acid hybridization and immunohistochemical techniques to demonstrate the presence and localization of the virus, as was critically important in delineating the role played by previous vertically transmitted viral diseases. 

All of the recent vertically transmitted emerging infections have had significant placental pathology findings identified that account for transplacental transmission. Placentas from pregnant women having COVID-19 have demonstrated a unique combination of findings termed SARS-CoV-2 placentitis, which include chronic histiocytic intervillositis, trophoblast necrosis, and massive fibrin deposition, as well as features of maternal vascular malperfusion, fetal vascular malperfusion, and hemorrhage [5,6,35,47]. In the only placenta examined from a fetus with congenital mpox syndrome, immunohistochemistry revealed that the chorionic villi had a large monkeypox virus (MPXV) viral load within a hyperplastic population of stromal macrophages (Hofbauer cells) [1,8,34]. Zika virus has been identified in transmitting placentas using immunohistochemistry and nucleic acid hybridization and causes Hofbauer cell hyperplasia [48]. In the few placentas examined from pregnant women having Ebola virus infection, Ebolavirus antigen was identified in the syncytiotrophoblast and maternal mononuclear cells in the intervillous space by immunohistochemical analysis [49].

Diagnostic reagents for demonstrating OROV in human pathology tissues are available [50]. It can be expected that, once a detailed examination of placentas obtained from fetuses having congenital OROV infection is performed, it will demonstrate any existing microscopic abnormalities from viral infection. In addition, the presence and anatomic distribution of this emerging bunyavirus may be able to be characterized using immunohistochemistry and/or in situ nucleic acid hybridization techniques, adding to our understanding of the mechanism for transplacental transmission and congenital infection.

## 7. Recommendations for Pregnancy

It is important to raise awareness among physicians in the Americas, Europe, and elsewhere regarding the newly identified potential for OROV to cause severe obstetrical and perinatal complications in infected mothers from endemic areas. Pregnant women, as well as any tourists, with OROV infection can rapidly be transported from endemic areas via international air travel during their viremic or post-viremic periods. They may not even be aware that they are infected or have a fetus at risk for congenital infection. Thus far, knowledge about Oropouche fever in pregnancy is limited to case reports and it remains unknown whether pregnant women are more vulnerable to infection or whether the disease has different clinical manifestations or levels of severity during pregnancy. Clinical case management of acute maternal illness is currently the same as in nonpregnant persons and includes such supportive measures as analgesia, fluids, antipyretic medications, and rest. When evaluating pregnant women for Oropouche fever it is important to exclude concomitant disease from dengue fever, chikungunya, and Zika virus infections, malaria, leptospirosis, and other infections from the endemic regions. There is no vaccine or antiviral medication that is effective for Oropouche fever. The role of amniocentesis in detecting OROV infection is unknown. For Zika virus, amniotic fluid and fetal blood remain positive only transiently, whereas maternal viremia might be prolonged in case of severe fetal infection [51]. Whether this is similar for OROV needs to be investigated. As discussed in this report, the finding of congenital transmission and fetal malformations associated with OROV means that a high index of suspicion should exist if pregnant women have a travel history to the areas of outbreak. Prenatal ultrasound for detection of microcephaly and brain abnormalities should be considered, similar to the situation that occurred during the Zika virus epidemic. However, there is currently insufficient information available to determine the optimum timing for an initial fetal ultrasonography [40]. It is currently recommended that pregnant women exposed to OROV have ultrasonographic screening at 4-week intervals with special attention to the fetal neuroanatomy [40]. As occurred during the Zika virus epidemic, pregnant women may wish to reconsider unnecessary travel to the areas affected by the epidemic having an OROV Level 2 Travel Alert [15]; if travel is essential, pregnant women should take appropriate precautions to limit exposure to the insect vectors [52,53]. These precautions would include wearing long-sleeve shirts and pants, being indoors in a protected area or a room with window and door screens, outdoor fans, and use of a DEET-containing insect repellent registered by the EPA [53]. It is highly recommended that consultation with an obstetrician or maternal–fetal medicine physician with expertise in congenital infections be considered to enhance early detection and management of travel-associated cases. 

Diagnostic testing methods for pregnant women exposed to OROV is the same as for nonpregnant persons. A preliminary diagnosis may be based upon travel history to an endemic area, activities leading to potential exposure, and clinical symptoms [15]. The virus can be detected in maternal serum samples during the initial week of infection, after which viral nucleic acid may be detectable using PCR. Following this, there is production of IgM antibodies to the virus and, later, IgG antibodies, which can be detected by serology. It is optimal to have both acute and convalescent serum samples available to detect a 4-fold increase in OROV antibody titers [15]. Recommendations for clinical testing for OROV infection are available from the CDC, which include performance of CLIA-validated plaque reduction neutralization tests (PRNT) that can identify virus-specific neutralizing antibodies that are usually present after the first week of OROV infection [53,54]. The CDC has an OROV rRT-PCR, which is non-CLIA-validated and expected to be available for testing in serum and cerebrospinal fluid specimens [55]. 

## 8. Conclusions

Oropouche fever is estimated to put as many as five million people throughout the Americas at risk for exposure, making it one of the most important emerging viral diseases in Latin America [56], which is supported by its recent association with adverse obstetrical outcomes. Based upon current data, including past outbreaks and the current 2023–2024 epidemic, pregnant women with Oropouche fever have had miscarriages, stillbirths, and newborns with microcephaly [14,18,30,31,32,38]. OROV has been definitively identified in pregnant women and their infants by molecular biological diagnosis in the placenta; umbilical cord; fetal somatic organs, including brain, kidneys, spleen, lungs, liver, heart, pleural fluid, and cerebrospinal fluid; antibodies in serum of pregnant mothers having poor perinatal outcomes; and antibodies in the cerebrospinal fluid and serum of neonates with microcephaly [18,32,39,40].

## Figures and Tables

**Table 1:** Fetal outcomes of recent epidemic emerging viruses.

Virus/Disease (Abbreviation, Genus)	Oropouche (OROV, Orthobunyavirus)	Mpox (MPXV, Orthopoxvirus)	COVID-19 (SARS-CoV-2, Betacoronavirus)	Zika (ZIKA, Flavivirus)	Ebola (EBOV, Ebolavirus)
Intrauterine congenital transmission	Yes	Yes	Yes	Yes	Yes
Placental infection	Yes	Yes	Yes	Yes	Yes
Miscarriage	Yes	Yes	Yes	Yes	Yes
Stillbirth	Yes	Yes	Yes	Yes	Yes
Preterm birth	Unknown	Yes	Yes	Yes	Yes
Microcephaly	Yes	No	No	Yes	No
Malformations	Yes	No	No	Yes	No
